# Respiratory Impairment after Early Red Cell Transfusion in Pediatric Patients with ALI/ARDS

**DOI:** 10.1155/2012/646473

**Published:** 2012-08-26

**Authors:** Surender Rajasekaran, Dominic Sanfilippo, Allen Shoemaker, Scott Curtis, Sandra Zuiderveen, Akunne Ndika, Michael Stoiko, Nabil Hassan

**Affiliations:** ^1^Division of Pediatric Critical Care Medicine, Helen DeVos Children's Hospital, 100 Michigan Street NE, Grand Rapids, MI 49503, USA; ^2^Research Department, Grand Rapids Medical Education Partners, 1000 Monroe NW, Grand Rapids, MI 49503, USA

## Abstract

*Introduction*. In the first 48 hours of ventilating patients with acute lung injury (ALI)/acute respiratory distress syndrome (ARDS), a multipronged approach including packed red blood cell (PRBC) transfusion is undertaken to maintain oxygen delivery. *Hypothesis*. We hypothesized children with ALI/ARDS transfused within 48 hours of initiating mechanical ventilation would have worse outcome. The course of 34 transfused patients was retrospectively compared to 45 nontransfused control patients admitted to the PICU at Helen DeVos Children's Hospital between January 1st 2008 and December 31st 2009. *Results*. Mean hemoglobin (Hb) prior to transfusion was 8.2 g/dl compared to 10.1 g/dl in control. P/F ratio decreased from 135.4 ± 7.5 to 116.5 ± 8.8 in transfused but increased from 148.0 ± 8.0 to 190.4 ± 17.8 (*P* < 0.001) in control. OI increased in the transfused from 11.7 ± 0.9 to 18.7 ± 1.6 but not in control. Ventilator days in the transfused were 15.6 ± 1.7 versus 9.5 ± 0.6 days in control (*P* < 0.001). There was a trend towards higher rates of MODS in transfused patients; 29.4% versus 17.7%, odds ratio 1.92, 95% CI; 0.6–5.6 Fisher exact *P* < 0.282. *Conclusion*. This study suggests that early transfusions of patients with ALI/ARDS were associated with increased ventilatory needs.

## 1. Introduction

Acute lung injury (ALI) and acute respiratory distress syndrome (ARDS) have high morbidity and mortality inflicting a substantial burden on families and healthcare institutions [1–3]. ALI can be triggered by pulmonary insults like pneumonitis or systemic disease such as sepsis or trauma. As ALI evolves, lung compliance deteriorates and ventilation/perfusion (V/Q) mismatch worsens, threatening oxygenation. There are consensus statements that recommend packed red blood cell (PRBC) transfusion of anemic critically ill patients with ARDS to augment volume expansion and oxygen delivery [4]. There continues to be the assumption that anemia is potentially more compromising to the critically ill child than a PRBC transfusion. Therefore, blood transfusions are given to the sickest patients early in the course of their illness, often when they are unstable and already requiring cardiopulmonary support. 

Data from the pediatric acute lung injury sepsis investigators (PALISI) network show that about 49% of pediatric intensive care unit (PICU) patients receive blood transfusion early, mostly within 48 hours of admission to the ICU [5]. There is also evidence that alveolar damage occurs as early as 48 hours into the course of ARDS [6, 7] and the highest surges in proinflammatory cytokines occur early in the development of the disease [8]. It is therefore reasonable to speculate that blood transfusions early in the course of ALI or ARDS may serve as a second insult which could further aggravate and propagate the inflammatory state associated with the evolution of ALI or ARDS. 

Specifically, we hypothesized that patients with ALI or ARDS who receive a blood transfusion in the early phase of mechanical ventilation might suffer a more complicated course which offsets any initial benefit. Therefore, in this retrospective study, we focused on the pediatric patients receiving PRBCs in the first 48 hours of mechanical ventilation comparing them with others who also met ALI or ARDS criteria but were not transfused.

## 2. Materials and Methods

Institutional review board approval was obtained and the need for informed consent waived because the research was considered to be of minimal risk. For a two-year period from January 2008 to December 2009, all arterial line placements in a tertiary care PICU at Helen DeVos Children's Hospital were identified. This allowed us to use PaO_2_ to FiO_2_ ratio to classify ALI/ARDS as outlined by the American European Consensus Conference (AECC). The clinical course of those patients were examined, with 107 patients identified as meeting ALI or ARDS criteria; only 34 transfused patients and 45 nontransfused met study criteria for inclusion. Twenty eight patients were excluded because they did not meet inclusion criteria; with the majority being related to timing of transfusion. In addition, the excluded patients were separately analyzed to determine their demographic characteristics and if any of these characteristics differed significantly from the studied population.

The decision to transfuse PRBCs was made by the PICU attending on service rather than by any predetermined protocol. It was based on clinical assessment of the patient's condition, the degree of anemia and determination that a transfusion would be beneficial. 

Various information databases including Virtual PICU Systems (VPS) and Cerner were utilized to determine clinical course.

**Study Group Time Points (Transfusion Group)**

Once intubated, ventilator settings, laboratory indices and scores based on blood gas tensions were recorded; the last set of values prior to blood transfusion was recorded as the “before value.” The next set of values was recorded 24 hours after transfusion and designated as “after transfusion values.” For both groups, the third time point was at 96 hours post initiation of mechanical ventilation.

**Control Group Time Points**

The last value on the first 24 hours of mechanical ventilation was compared with the “before values” of the study group. The last value of the first 48 hours of mechanical ventilation was compared with the “after values” of the study group. For both groups, the final outcomes and incidence of organ dysfunction later in the course of mechanical ventilation were noted.

### 2.1. Inclusion Criteria

- All patients <18 years of age that met ALI or ARDS diagnostic criteria as outlined by the AECC within the first 48 hours of mechanical ventilation.
- Transfusion of PRBCs within 48 hours of initiating mechanical ventilation (study group).
- No blood transfusion within the first 48 hours of mechanical ventilation (control group).

### 2.2. Exclusion Criteria

- Patients meeting diagnostic criteria for pulmonary hypertension.
- Active bleeding from trauma or surgery.
- Patients with preexisting multiple organ dysfunction syndrome (MODS) at intubation.
- Patients who received plasma or platelets in the first 48 hours of mechanical ventilation.
- Patients on High frequency oscillatory ventilation (HFOV) were excluded from the study because the ventilator parameters were not comparable to conventional ventilation.
- Any patient in either group requiring extracorporeal support (continuous renal replacement therapy (CRRT) or extracorporeal membrane oxygenation (ECMO)) in the first 48 hours of mechanical ventilation.
- Patients receiving PRBC transfusion prior to initiating mechanical ventilation.

### 2.3. Measured Indices

PaO_2_/FiO_2_ (P/F) ratios and Oxygenation Indices (OI) = (FiO_2_∗Mean Airway Pressure∗100/PaO_2_) obtained for the time periods described above; number and type of organ system failures, length of mechanical ventilation, and survival at PICU discharge were recorded.

The primary diagnoses requiring invasive mechanical ventilation in patients with ALI/ARDS were categorized as follows: pneumonia, aspiration pneumonia, sepsis, trauma, and others. The etiology for ALI/ARDS as direct injury or indirect injury was recorded. 

The PRISM III scores were determined for the first 12 hours of admission. All patients had chest radiographic findings immediately after intubation and then daily which were used to establish ALI/ARDS diagnosis. Ventilator indices, PaO_2_/FiO_2_ ratios and OI were determined based on the arterial blood gas assessed 6–12 hrs after intubation to allow for lung recruitment and establishment of relatively steady state pulmonary function [9]. 

The American European Consensus Conference criteria were used to diagnose ALI/ARDS. This utilizes four clinical parameters: (a) acute onset, (b) severe arterial hypoxemia resistant to oxygen therapy alone (PaO_2_/FiO_2_ ratio ≤200 torr (≤26.6 kPa) for ARDS and PaO_2_/FiO_2_ ratio ≤300 torr (≤40 kPa) for ALI), (c) diffuse pulmonary inflammation (bilateral infiltrates on chest radiograph), and (d) no evidence of left atrial hypertension [10]. Organ system failures were defined according to the criteria established by Wilkinson et al. [11] and subsequently modified by the American College of Chest Physicians/Society of Critical Care Medicine Consensus Conference [12]. 

Percent fluid balance was calculated for the 24 hours before and the 24 hours after initiation of mechanical ventilation based on the formula described by Goldstein et al.: ((Fluid in-Fluid out (L))/(admission weight (kg)) ×100) [13]. The volume of blood given and the age of the blood were noted.

The primary outcome variables were: (1) P/F ratios and OI at 48 hours post initiation of mechanical ventilation and (2) ventilator days. 

### 2.4. Statistical Methods

Descriptive statistics were generated and expressed as: means ± SEM (standard error). *t*-test was used to compare the means of all continuous variables comparisons. Pearson chi-square test was used to determine any relationship between categorical variables. This was followed with odds ratio testing wherever appropriate. Logistic regression was done to test for interactions between some risk factors. All data analyses were done using SPSS software (V. 17, 2008).

## 3. Results

Thirty-four patients with ALI and ARDS were transfused and their clinical course was compared to forty-five patients who served as controls. The time interval from initiation of mechanical ventilation to time point was at which laboratory tests were done, was separately analyzed to determine congruity of time points. The lab values from the “before transfusion” time point were on average 19.6 ± 4.2 hours from initiation of mechanical ventilation for transfused patients compared to values of 22 ± 5.1 hours for control (*P* < 0.776). The “after transfusion” value averaged at 56.1 ± 3.1 hours from initiation of mechanical ventilation for the transfused group compared to 49.7 ± 6.2 for control (*P* < 0.566).

### 3.1. Demographic Variables (Table 1)

The average age of the transfused group tended to be younger, 48.7 ± 12.3 months compared to 72.3 ± 9.6 months for control (*P* < 0.129). PRISM III scores were comparable (11.8 ± 3.2 versus 9.2 ± 4.2 in control) (*P* < 0.587). The other demographic characteristics of both groups are reported in Table 1. The 15 excluded transfused patients were separately analyzed and compared with those included; majority excluded because of PRBC transfusion out of the range, 46.6% (7 of 15), pre-existing MODS, 20% (3 of 15), HFOV, 20% (3 of 15), and 13.3% (2 of 15) received other blood products.

Demographic data such as age, weight, gender, PRISM III score and pre-Hb were compared and analyzed. Only PRISM score in included 11.8 ± 3.2 versus 15.8 ± 3.8 for excluded patients was significantly different (*P* < 0.038). The 13 control patients were not separately analyzed because the numbers were thought to be too few. The majority of those were patients with preexisting MODS 38.5% (5 of 13).

### 3.2. Transfusion Parameters

Thirty-four patients were transfused with leukodepleted PRBCs, receiving an average volume of 13.9 ± 3.2 mL/kg. Transfusions were given for mean hemoglobin of 8.2 ± 0.2 g/dL and increased hemoglobin to 11.0 ± 0.2 g/dL compared to 10.1 ± 0.2 g/dL on day 1 for control. The indications were anemia for 19 patients, hemodynamic instability for 13 patients, and hypoxia for 2 patients. Six of 34 (17.1%) patients received intravenous furosemide after receiving packed cells. Median storage age of the blood was 19 days (range 2–34).

*Logistic Regression* was done to assess the cumulative impact of risk factors which were predictive of a transfusion occurring. Transfusion was associated with negative fluid balance (*P* < 0.002), pretransfusion Hb (*P* < 0.001) and pre-P/F ratio (*P* < 0.025) but not PRISM III score (*P* < 0.580) or vasopressor use (*P* < 0.667).

### 3.3. Oxygenation, Ventilator, and Other Indices

On average, transfused patients spent 5 more days on the ventilator; 15.2 ± 1.4 days compared to 9.5 ± 0.6 days in controls (*P* < 0.000) (Table 2). Baseline P/F ratios in both groups did not differ (135.4 ± 7.5 for transfused versus 148.0 ± 8.0 for controls (*P* < 0.922)). After 48 hours of mechanical ventilation, the P/F ratio was 116.5 ± 8.8 in transfused compared to 190.4 ± 17.8 in control (*P* < 0.001). 

OI increased from 11.7 ± 0.9 to 18.7 ± 1.6  (*P* < 0.004) after PRBC infusion; whereas in control, OI decreased from 12.3 ± 0.8 to 11.1 ± 0.9  (*P* < 0.342). Between groups, there was a difference in OI in the first 48 hours (*P* = 0.001), and no significant difference in OI or P/F ratio noted at 96 hours. Transfused patients had a PaCO_2_ of 48.6 ± 1.6 torr which increased to 57.2 ± 1.5 torr after PRBC (*P* < 0.012), whereas in control, the change was 51.1 ± 2.2 torr to 53 ± 2.7 torr (*P* < 0.402) over a comparable period. The other pulmonary variables of interest are described in Table 2.

### 3.4. Hemodynamic Parameters

Fluid overload was 7.6 ± 0.9% in control and 4.3 ± 1.1% in transfused patients. Seven patients in the transfused group received furosemide compared to only two in the control group. Within the transfused group, the seven who received diuretics had a significantly negative fluid balance of −1.4% compared to the non-diuresed transfused who had a fluid balance of +6.0% (*P* < 0.03).

In the transfused group, 5.8% (2 of 34 patients) had a decrease in the need for vasopressor therapy. The rest had either no change in vasopressor dose; 8 of 34 (23%), or increase; 3 of 34 (8.8%). Over comparable time points in the control group, 6 of 45 patients (13.3%) needed a vasoactive infusion after 24 hours of mechanical ventilation. 

### 3.5. Clinical Course

The transfused group had slightly more patients with MODS not reaching significance posttransfusion, with 29.4% (10 of 34) versus 17.7% (8 of 45) in control, odds ratio 1.92, 95% CI; 0.6–5.6, Fisher exact (*P* < 0.282). Most of this was related to transfused patients having more renal dysfunction with need for renal replacement therapy; 17.6% (6 of 34) compared to control 4.4% (2 of 45) (*P* < 0.057). Mortality was analyzed separately with 11.7% (4 of 34) patients in the transfused group not surviving their ICU course compared to 2.2% (1 of 45) in the control group. 

*Logistic Regression* was done to assess the contribution of multiple risk factors to MODS and neither PRISM III (*P* < 0.846) nor age of blood (*P* < 0.931) contributed to risk for MODS in our study.

## 4. Discussion

ALI and ARDS are types of restrictive lung disease characterized by surfactant dysfunction and parenchymal inflammation often leading to poor compliance and loss of functional residual capacity (FRC). To maintain oxygenation, clinicians employ high levels of FiO_2_ and airway pressures which often exacerbate lung inflammation, and impede venous return and cardiac output [14]. Indeed, some authors have recommended PRBC transfusions to maintain hemoglobin thresholds when tissue oxygenation and perfusion are poor to putatively improve oxygen delivery [4, 15]. Yet, there is increasing evidence that transfusions can have adverse effects in the critically ill patient such as increasing the incidence of MODS and ventilator days [16–18]. 

We found significant negative associations of PRBC transfusion in two main areas: acute lung function and ventilator days. There was also a trend towards higher rates of MODS with renal dysfunction requiring CRRT. About 24 hours after blood transfusion, those patients had a 39% lower P/F ratio and 38% higher OI than controls (Table 2). In the first 48 hours, PEEP was increased in both groups but the transfused group did not show the improvement in P/F ratio or OI noted in the control group. These differences diminished but were still 10.5% and 25.1% more than the control, respectively, at 96 hours, though not significant. Transfused patients had a 17% increase in PaCO_2_ after receiving packed cells (*P* < 0.012). They also had longer ventilator courses, with a mean of 15.2 ± 1.4 days versus controls with 9.5 ± 0.6 days (*P* < 0.001).

Our study supports the notion that blood transfusions have potential to complicate the early care of patients with ALI/ARDS as they required higher ventilator settings initially, despite not being significantly fluid overloaded. Due to the limitations imposed by the retrospective design, one can only speculate on the etiology of the mechanisms responsible for the clinical deterioration in our patients.

Transfusion related acute lung injury (TRALI) remains the best known phenomenon related to deterioration in lung function after transfusion. There are two distinct presentations of TRALI. First, the more classical occurs in patients who do not have an underlying pulmonary dysfunction or the risks associated with developing such [19]. Second, a more insidious delayed form, which has been better described in adults, occurring within 24 hours of blood transfusion in patients who already had an underlying risk for or presence of pulmonary dysfunction [20]. In expounding this type, Silliman et al. hypothesized that this form of TRALI, like ALI and ARDS, is the result of at least two separate clinical insults; the first is related to the clinical condition of the patient that causes activation of the pulmonary endothelium, leading to the sequestration of polymorphonuclear leukocytes (PMNs) to the activated pulmonary endothelium [21]. These activated PMNs release both oxidative and nonoxidative components. The second event is the infusion of specific antibodies directed against surface antigens on the PMNs and biological response modifiers in the stored blood that activate these primed, hyperactive PMNs, precipitating endothelial damage and capillary leak potentially worsening pulmonary compliance. This two insult pathway could be responsible for the clinical deterioration noted in our transfused patients. 

The tendency for transfused patients to develop MODS has been shown by other studies [18]. Recently, a study by Gauvin et al. showed that a blood storage time of more than 14 days was independently associated with increased MODS (adjusted OR, 2.23; 95% CI, 1.20–4.15) and storage time of more than 21 days was associated with increased pediatric logistic organ dysfunction (PELOD) scores and higher mortality [16]. There was a tendency for our transfused patients to develop MODS, mostly renal dysfunction requiring CRRT. The median age of blood utilized in our study was 19 days and did not associate with MODS. 

This study with its retrospective design has important limitations such as the small size and clinical diversity of the patients. Our intent was to study two groups that were comparably sick prior to transfusion; there remains the distinct possibility that transfused patients were sicker to start with and therefore suffered a more complicated course. Also, in order to conduct meaningful comparisons of ventilator parameters and clinical course between the two groups, certain groups were excluded such as patients on HFOV and those with MODS prior to intubation. Furthermore, there were patients who probably would have met ALI and ARDS criteria but did not have an arterial line and therefore could not be categorized. All of these factors could have contributed a certain amount of imbalance to sample selection and potentially modified the severity of illness in either studied group. 

## 5. Conclusions

Despite those limitations, the study population remains reflective of most PICU patients with lung injury. This study suggests that early attempts to improve tissue oxygenation using PRBC transfusions in the sickest pediatric patients can be associated with worse lung function and delayed recovery. Further studies need to be designed not only to establish the effect by using large-scale prospective design but should also include more focused studies examining the pathologic basis for the transfusion associated lung injury in critically ill patients after onset of respiratory failure.

## Figures and Tables

**Table 1:** Demographics of ALI and ARDS patients.

Indices	Transfused	Control	*P* value
Total patients	34	45	
Age (months)	48.7 ± 12.3	72.3 ± 9.6	0.129
Weight (kg)	19.6 ± 3.1	25.4 ± 4.6	0.276
Males	35.3%	57.7%	0.040
ARDS/ALI	29/5	36/9	0.377*
PRISM III score	11.8 ± 3.2	9.2 ± 4.2	0.587
Diagnostic criteria			
Sepsis	7 (20.6%)	15 (33.3%)	0.159
Pneumonia	17 (50%)	18 (40%)	0.255
Aspiration pneumonia	4 (11.7%)	6 (13.3%)	0.447
Trauma	3 (8.8%)	3 (6.6%)	0.472
Others	3 (8.8%)	3 (6.6%)	0.472
Indirect lung injury	13/34 (38.2%)	21/45 (46.6%)	0.301
Direct lung injury	21/34 (61.8%)	24/45 (54.4%)	0.301

Chi-square was done to determine any significance in relative proportion of variables per categorization.

*Statistic only for ARDS patients.

**Table 2:** Pulmonary indices.

Indices	Transfused	Control	*P* value
P/F ratio pre	135.4 ± 7.5	148.0 ± 8.0	0.922
P/F ratio post	116.5 ± 8.8	190.4 ± 17.8	0.001
P/F ratio 96 hr	178.4 ± 14.8	199.8 ± 14.6	0.332
OI pre	11.7 ± 0.9	12.3 ± 0.8	0.590
OI post	18.7 ± 1.6	11.1 ± 0.9	0.001
OI 96 hr	12.87 ± 1.3	10.31 ± 0.94	0.302
% change in MAP	+27.3	+14.3	0.165
Ventilator days	15.2 ± 1.4	9.5 ± 0.6	0.000
Median PEEP 24 hrs later	10 Range (5–15)	9 Range (5–14)	0.241

P/F: PaO_2_/FiO_2_, OI: oxygenation index (FiO2∗MAP∗100/PaO_2_), MAP: mean airway pressure. *t*-test was done to compare transfused and control values.

## References

[1] Bernard GR (2005). Acute respiratory distress syndrome: a historical perspective. *American Journal of Respiratory and Critical Care Medicine*.

[2] Dahlem P, van Aalderen WMC, Bos AP (2007). Pediatric acute lung injury. *Paediatric Respiratory Reviews*.

[3] Erickson S, Schibler A, Numa A (2007). Acute lung injury in pediatric intensive care in Australia and New Zealand—a prospective, multicenter, observational study. *Pediatric Critical Care Medicine*.

[4] Dellinger RP, Levy MM, Carlet JM (2008). Surviving sepsis campaign: international guidelines for management of severe sepsis and septic shock: 2008. *Critical Care Medicine*.

[5] Bateman ST, Lacroix J, Boven K (2008). Anemia, blood loss, and blood transfusions in North American children in the intensive care unit. *American Journal of Respiratory and Critical Care Medicine*.

[6] Fein AM, Grant MM, Niederman MS, Kantrowitz N (1991). Neutrophil-endothelial cell interaction in critical illness. *Chest*.

[7] Hammerschmidt DE, Vercellotti GM (1987). Granulocytes as mediators of tissue injury in shock: therapeutic implications. *Progress in Clinical and Biological Research*.

[8] Parsons PE, Eisner MD, Thompson BT (2005). Lower tidal volume ventilation and plasma cytokine markers of inflammation in patients with acute lung injury. *Critical Care Medicine*.

[9] Trachsel D, McCrindle BW, Nakagawa S, Bonn D (2005). Oxygenation index predicts outcome in children with acute hypoxemic respiratory failure. *American Journal of Respiratory and Critical Care Medicine*.

[10] Bernard GR, Artigas A, Brigham KL (1994). The American-European consensus conference on ARDS: definitions, mechanisms, relevant outcomes, and clinical trial coordination. *American Journal of Respiratory and Critical Care Medicine*.

[11] Wilkinson JD, Pollack MM, Ruttimann UE, Glass NL, Yeh TS (1986). Outcome of pediatric patients with multiple organ system failure. *Critical Care Medicine*.

[12] Proulx F, Fayon M, Farrell CA, Lacroix J, Gauthier M (1996). Epidemiology of sepsis and multiple organ dysfunction syndrome in children. *Chest*.

[13] Goldstein SL, Currier H, Graf JM, Cosio CC, Brewer ED, Sachdeva R (2001). Outcome in children receiving continuous venovenous hemofiltration. *Pediatrics*.

[14] Kobr J, Fremuth J, Pizingerova K (2011). Repeated bedside echocardiography in children with respiratory failure. *Cardiovascular Ultrasound*.

[15] Randolph AG (2009). Management of acute lung injury and acute respiratory distress syndrome in children. *Critical Care Medicine*.

[16] Gauvin F, Spinella PC, Lacroix J (2010). Association between length of storage of transfused red blood cells and multiple organ dysfunction syndrome in pediatric intensive care patients. *Transfusion*.

[17] Karam O, Tucci M, Bateman ST (2010). Association between length of storage of red blood cell units and outcome of critically ill children: a prospective observational study. *Critical Care*.

[18] Marik PE, Corwin HL (2008). Efficacy of red blood cell transfusion in the critically ill: a systematic review of the literature. *Critical Care Medicine*.

[19] Popovsky MA, Abel MD, Moore SB (1983). Transfusion-related acute lung injury associated with passive transfer of antileukocyte antibodies. *American Review of Respiratory Disease*.

[20] Gajic O, Rana R, Winters JL (2007). Transfusion-related acute lung injury in the critically Ill: prospective nested case-control study. *American Journal of Respiratory and Critical Care Medicine*.

[21] Silliman CC, Voelkel NF, Allard JD (1998). Plasma and lipids from stored packed red blood cells cause acute lung injury in an animal model. *The Journal of Clinical Investigation*.

